# Machine Gaze: Self-Identification Through Play With a computer Vision-Based Projection and Robotics System

**DOI:** 10.3389/frobt.2020.580835

**Published:** 2020-12-17

**Authors:** RAY LC, Aaliyah Alcibar, Alejandro Baez, Stefanie Torossian

**Affiliations:** ^1^School of Creative Media, City University of Hong Kong, Kowloon, Hong Kong; ^2^New York Hall of Science, New York, NY, United States; ^3^Department of Mechanical Engineering, New York University, New York, NY, United States; ^4^Department of Art, Queens College City University of New York, New York, NY, United States

**Keywords:** robotic art, human machine communication technology, projection mapping, computer vision, human robot interaction, child psychology, self-identify

## Abstract

Children begin to develop self-awareness when they associate images and abilities with themselves. Such “construction of self” continues throughout adult life as we constantly cycle through different forms of self-awareness, seeking, to redefine ourselves. Modern technologies like screens and artificial intelligence threaten to alter our development of self-awareness, because children and adults are exposed to machines, tele-presences, and displays that increasingly become part of human identity. We use avatars, invent digital lives, and augment ourselves with digital imprints that depart from reality, making the development of self-identification adjust to digital technologies that blur the boundary between us and our devices. To empower children and adults to see themselves and artificially intelligent machines as separately aware entities, we created the persona of a salvaged supermarket security camera refurbished and enhanced with the power of computer vision to detect human faces, and project them on a large-scale 3D face sculpture. The surveillance camera system moves its head to point to human faces at times, but at other times, humans have to get its attention by moving to its vicinity, creating a dynamic where audiences attempt to see their own faces on the sculpture by gazing into the machine's eye. We found that audiences began attaining an understanding of machines that interpret our faces as separate from our identities, with their own agendas and agencies that show by the way they serendipitously interact with us. The machine-projected images of us are their own interpretation rather than our own, distancing us from our digital analogs. In the accompanying workshop, participants learn about how computer vision works by putting on disguises in order to escape from an algorithm detecting them as the same person by analyzing their faces. Participants learn that their own agency affects how machines interpret them, gaining an appreciation for the way their own identities and machines' awareness of them can be separate entities that can be manipulated for play. Together the installation and workshop empower children and adults to think beyond identification with digital technology to recognize the machine's own interpretive abilities that lie separate from human being's own self-awareness.

## Background

### Development of Self-Awareness

The maxim of “Know thyself” has been touted since the time of Protagoras, as it indicates ultimate understanding of our own identity and action that allows us to more objectively evaluate our influence on the world. Recognition of self-awareness and self-identity fosters understanding of our relation to ourselves and our society as children and adults. Experiments show that the affirmation that comes with self-awareness leads to increased compassion for one's own actions as well as increased positive social helping behavior following surprising incidents like an accidentally collapsing shelf (Lindsay and Creswell, 2014). Self-awareness increases the attribution of causality for negative consequences to the self (Duval and Wicklund, 1973), serving to deter blaming others and deflecting criticism. Publically suggesting self-awareness using a webcam reduces the bystander effect of not helping someone in need when other people are present (van Bommel et al., 2012). Self-awareness induced by a mirror even reduces aggressive action, whereas audience presence does not (Scheier et al., 1974). Thus, self-awareness and identity go hand-in-hand with socially positive behaviors that promote integration in society.

The development of self-awareness and identity in children occurs in systematic stages that are often assayed using their response to seeing themselves in a mirror. Throughout the course of 5 years after birth, children go through eras of confusion, differentiation, identification, and meta-awareness in interactions with a mirror, characterized by what they do with their own bodies and objects placed in conjunction to them, such as post-its attached to their heads (Rochat, 2003). The last awareness stage involves how they present themselves publically, as if imagining how the mirror can be projected in the mind of others (Goffman, 1959). From 6 to 10 years old, children begin to consider alternatives to their own identities and at 10 years old, can even consider that their personalities remain the same when the name is taken away (Guardo and Bohan, 1971). This suggests at this age, children begin incorporating awareness of another viewpoint's perspective into their own self-awareness (Mitchell, 1993). This development is thought to occur in conjunction with biofeedback from parents, who present a reflective view for the children much like a mirror does in regulating their affective states (Gergely, 1996). Children begin to understand themselves by seeing the way others see them. In particular, the awareness of not being seen gives rise to an identification of the self as apart from the others' gaze.

Self-awareness adaptation doesn't end with childhood. Reflexivity in social interactions in considering one's own current and past selves allows emerging adults to construct their self-identity in the counseling setting (Guichard et al., 2012). Self-awareness is also crucial in leadership development (Hall, 2004) and promoting well-being in jobs such as mental health professionals (Richards et al., 2010). Public self-awareness of adults in a controlled interaction is found to predict variables like social anxiety, self-esteem, and perception of others (Ryan et al., 1991), indicating its importance in determining self-competence and social success. This self-identity in adults is bound up with bodily awareness. Those who lose bodily awareness due to trauma or injury are ameliorated using self-awareness-based touching and performance in psychological contexts (Fogel, 2009).

### Technologies for Self-Awareness

Getting good at theater and dramaturgy involves comparing one's actions to the action's perception, as well as collaborating together with other performers. This has led to the use of ideas from theater in teaching strategies for self-development. Studies have used collaborative theatrical projects to empower youths in such areas as creating meaning about the self (Beare and Belliveau, 2007), learning to improvise in hypothetical situations (Lehtonen, 2012), and achieving positive mental health (Ennis and Tonkin, 2015). One approach uses puppetry to enact fear, anger, sadness, and other emotion-based stories as part of a “feelings curriculum” to teach emotional awareness and self-comprehension to children (Maurer, 1977; LC, 2019). These traditions leverage the way theater forces individuals to reflect back on themselves upon identifying with actors in a scene. One system engages youths to use Twitter posts to emotionally affect physical actions of a puppet theater installation using a robotic arm in a video, allowing them to reflect on their communication for development of self-awareness (Yamaguchi, 2018). Essentially theater serves as an immersive version of a mirror that allows young people to gaze at their own actions and consequences as compared to those of others, driving a deeper meaning of what constitutes self-identity in the context of self-presentation. In particular, youths learn that social interactions involve presenting themselves in different ways in different contexts, much as actors play their roles in dramaturgy (Goffman, 1959). The practice of this self-presentation is made possible by both understanding the consequences of our own actions, and observing how others see us through their own lenses.

Interactive technologies for development of self-awareness have focused on vulnerable populations who have difficulty adjusting to societal norms due to their deficits in self-awareness, such as those suffering from communication and social disorders like autism and ADHD (Boucenna et al., 2014). Therapeutic strategies have included using touched-based devices to engage youths to foster development (Kagohara et al., 2013), applying virtual environments (such as VR cafes and buses) to allow youths to apply their social awareness skills incrementally without fear (Mitchell et al., 2007), creating serious games that effectively teach facial recognition in social situations (Serret, 2012), and utilizing social media platforms to enhance self-esteem by the way of profile identification (Gonzales and Hancock, 2010). Digital technologies of human-computer communication have been found to higher levels of private self-awareness compared to face-to-face communication, which heightened public self-awareness (Matheson and Zanna, 1988).

Of the various forms of communication technology, one of the most promising is robotics, for it enables physical interaction in addition to virtual ones at a distance. Early studies focused on using robots to imitate child action, generating a sequence of motor actions that reproduces a detected human gesture (Berthouze et al., 1996). This work has modeled social interaction as observation followed by motor control, producing statistical models of motor representations that attempt to capture the human-robot interaction, exemplified by a study utilizing a game played by the robot Vince and its human interlocutor (Sadeghipour and Kopp, 2011). While simple actions can be approximated by robot movements, complex interactions that involve environmental constraints and rules require applying statistical learning theory to average over the different possibilities in complex spaces for all possible movements, even in tasks as seemingly simple as putting objects into a box (Hersch et al., 2008). Recent work has modeled interactive tasks like tossing and catching arbitrary objects using both physics and computer vision to adaptively learn and generalize complex tasks (Zeng et al., 2020). One important contribution of related work is showing that using a game involving imitation with each other, human and robot become involved in feedback loops of reciprocal imitation, relying on human recognition and awareness on one hand and robot pose detection on the other (Boucenna et al., 2012). This begs the question of whether using simpler technologies like face detection is sufficient to elicit rich interactions that rely on human understanding rather than on complexity on the robotics side.

The use of robotics to elicit behaviors in human participants relies more on a rich interaction environment as opposed to a sophisticated computer vision detection model, due to the way humans are innately drawn to interpret even simple machine gestures as representing affective gestures analogous to human emotional behaviors (Sirkin et al., 2015; Knight et al., 2017; LC, 2019). Robots in this regard have taken such simple forms such as bubble-blowing agents (Feil-Seifer and Matarić, 2009), geospatial robots (Nugent et al., 2010), and gaze-directing toys (e.g., My Keepon) (Kozima et al., 2007), all using simple interactions utilizing remote control of robot interactions to promote pro-social behavior. The effectiveness of the strategy comes not from the intricacy of the interaction, but rather the rich set of environmental cues and interpretations available to the child that makes the experience rewarding. One way to increase the interaction and immersion in the physical environment is by augmenting it with strategies like projection (Greene, 1986). Recent work has been able to projection map custom imagery onto complicated forms like faces (Bermano et al., 2017) and moving objects (Zhou et al., 2016), opening up possibilities for single-object projection experiences that respond to human interaction. It is possible to map robotic responses onto interactive objects much like an immersive form of computer based sculpture (Keskeys, 1994). The projection would then provide an interface to the robot via an external material, adding an additional layer of interaction capabilities as if the robot is controlling the external visual interaction based on audience feedback.

### General Approach

Given the considerations above, we decided to use the robot's own interpretive ability—its gaze—to show young audiences the process of self-awareness, allowing them to understand themselves by seeing the way machine sees them. We used a simple face detection interaction with a moving robot to engage young audiences to become aware of the self through looking at themselves on a responsive projection mapped face sculpture, relying on the innate human ability to interpret the interaction environment in an affective manner.

This approach leverages: (1) the way children learn of self-awareness through the way others see them (Mitchell, 1993), (2) the physical proxemics and performance-like interactions that robotics creates to make this learning embodied in the real world, (3) the richness in self-gaze-directed interactivity provided by environmental augmentation through the mirror-like projected sculpture, and (4) the collaborative learning and play through workshops in multiple media and perspectives.

## Materials and Methods

The experience consisted of the following main components: (1) a motorized security-camera-like robot that moves either casually on its own or in response to audiences to keep its gaze on a face in the crowd, (2) a projection system that maps an audience member's own face onto a 3D face sculpture whenever the audience's face is detected by the robot, (3) a feedback screen that allows audiences to see what the machine is seeing, i.e., whether a face is detected, to interpret the machine's awareness of the audience, and (4) a workshop where audiences are asked to escape the machine's detection by putting on disguises, showing a comparison of being seen vs. not being seen by the machine, demonstrating a difference in awareness by other entities.

### Exhibition

**Figure 1:**
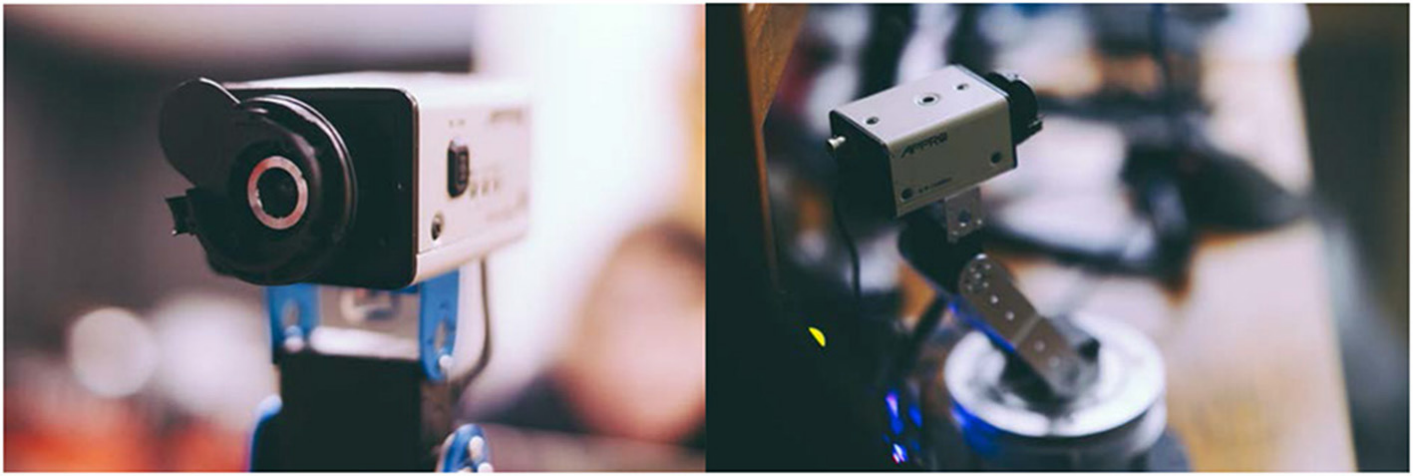
Robot head and body. **(Left)** The camera (head) was an APPRO model with lens and circuit replaced by a PC-connected webcam, mounted on steel plates. **(Right)** The body consisted of a steel frame joined by servo motors exhibiting three degrees of freedom, two of tilt and one of rotation, allowing the camera to face any direction in space.

A set of four Appro and Panasonic CP414 security cameras (circa 1980) were cleaned, refurbished, and mounted on metal plates. Two of the cameras were further chosen for prototyping, with their internal fisheye cameras removed and replaced by webcams connected to an Intel NUC 7 (Windows 10) mini computer. The internal circuit was taken out, and the lens chassis was then reattached over the webcam. The body of the robot was constructed from a rotating base plate and an arm that tilts up and down at two different joints (Lewansoul kit), spray-painted silver upon completion. The three degrees of freedom (one in rotation, two in tilt) were controlled using three LDX-218 servo motors connected to a controller board, which was interfaced to an Arduino UNO board using custom routines. Figure 1 shows the look of the camera and body, which were designed to appeal to young audiences, evoking playfulness and a perception of simplicity as opposed to traditional mechanized robots. The movements of the robot were similarly designed for serendipity, as sometimes the robot moved to fix its gaze on a face of the audience member, while other times it simply moved side to side and up and down on its own. The video stream taken by the webcam was processed in Processing 3.3 using OpenCV (Viola and Jones, 2001). During the face tracking phase, distance from the center of the view to the center of the detected face was calculated live, and whenever the x or y distance was non-zero, a signal was sent from Processing to Arduino to move the appropriate motors in that dimension to point the camera directly at the center of the audience's face. When multiple faces were detected, the robot would direct itself at each face in succession after a one-second pause in position. At other times, a set of three predetermined movement routines had the robot scanning around the exhibition hall. Occasionally, the robot would also move its head forward or backward toward imaginary objects. To appeal to younger audiences, we created a narrative for the robot as a supermarket surveillance camera fortified with computer vision and repurposed to play and teach children about machine gaze and self-recognition.

**Figure 2:**
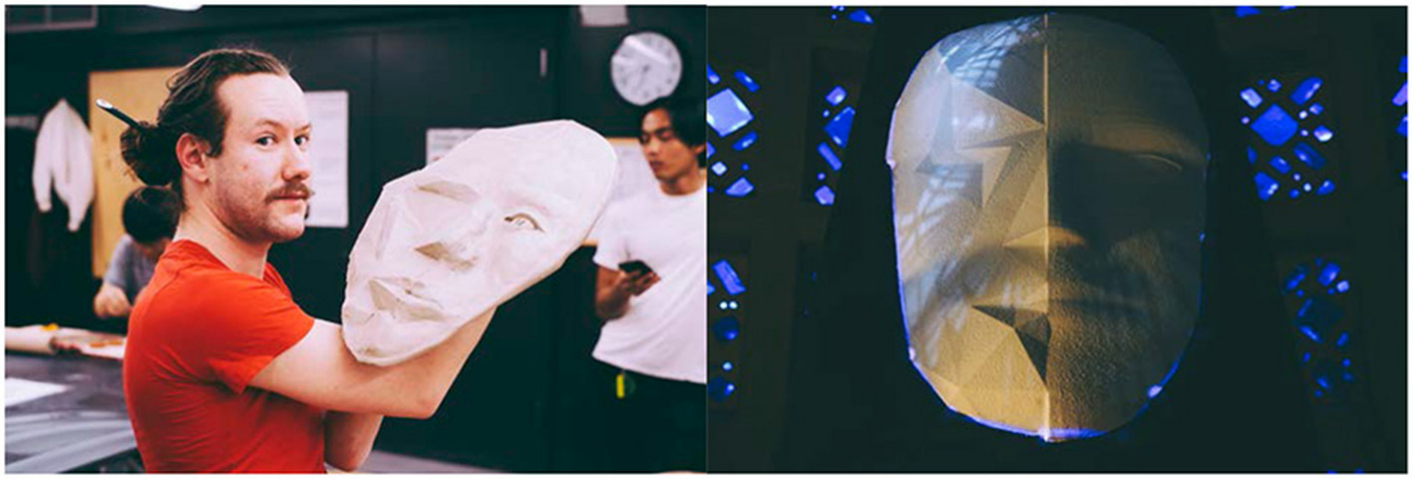
Prototypes of the 3D face sculpture. **(Left)** A clay model with right side sculpted to be human face and left side a polygonal surface. The size required turned out to be prohibitively heavy. **(Right)** A reduced-size foamular model cut by CNC from an stl model and painted white to properly reflect projected image. The final exhibition model was approximately twice times the width and twice the height.

**Figure 3:**
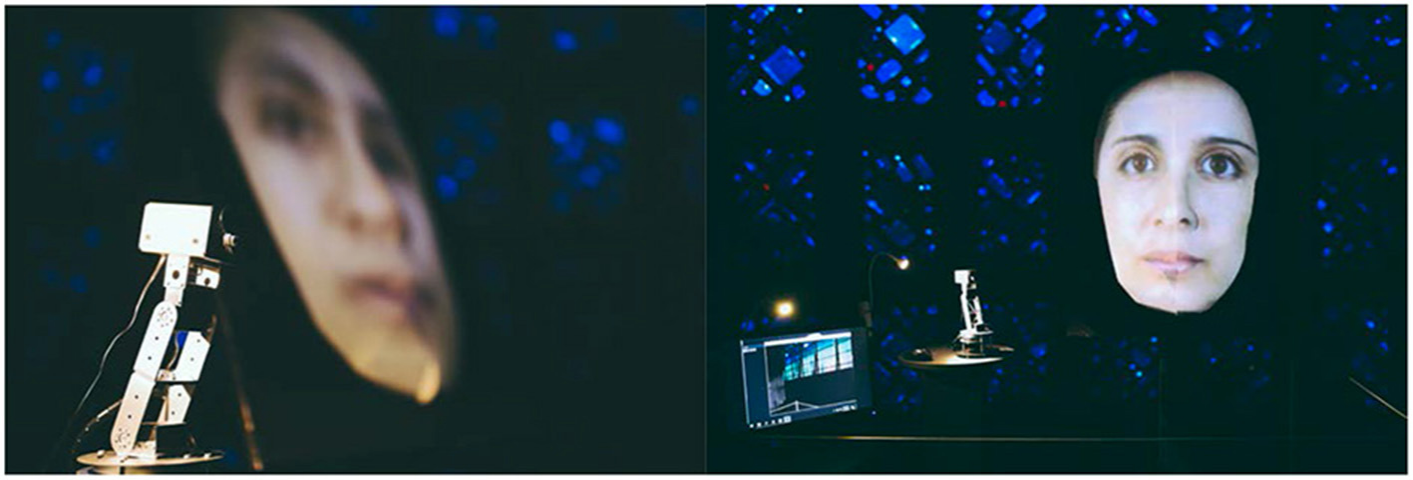
The exhibition setup. **(Left)** The camera-mounted robot sat on a dark-colored podium to the left of the face sculpture with the image of a face projected on it from approximately a 40° angle. **(Right)** The setup as viewed from an approaching audience, with a screen on the left showing the camera view from the perspective of the robot, and giving feedback to participants for when their faces were detected. One lamp lit the robot while the other lamp provided ambient lighting on the audience's face. The projected video on the face sculpture cycled between faces from the Chicago Face Database when no audience faces were detected, and a scaled version of the audience's face when it is detected by the webcam on the robot.

**Figure 4:**
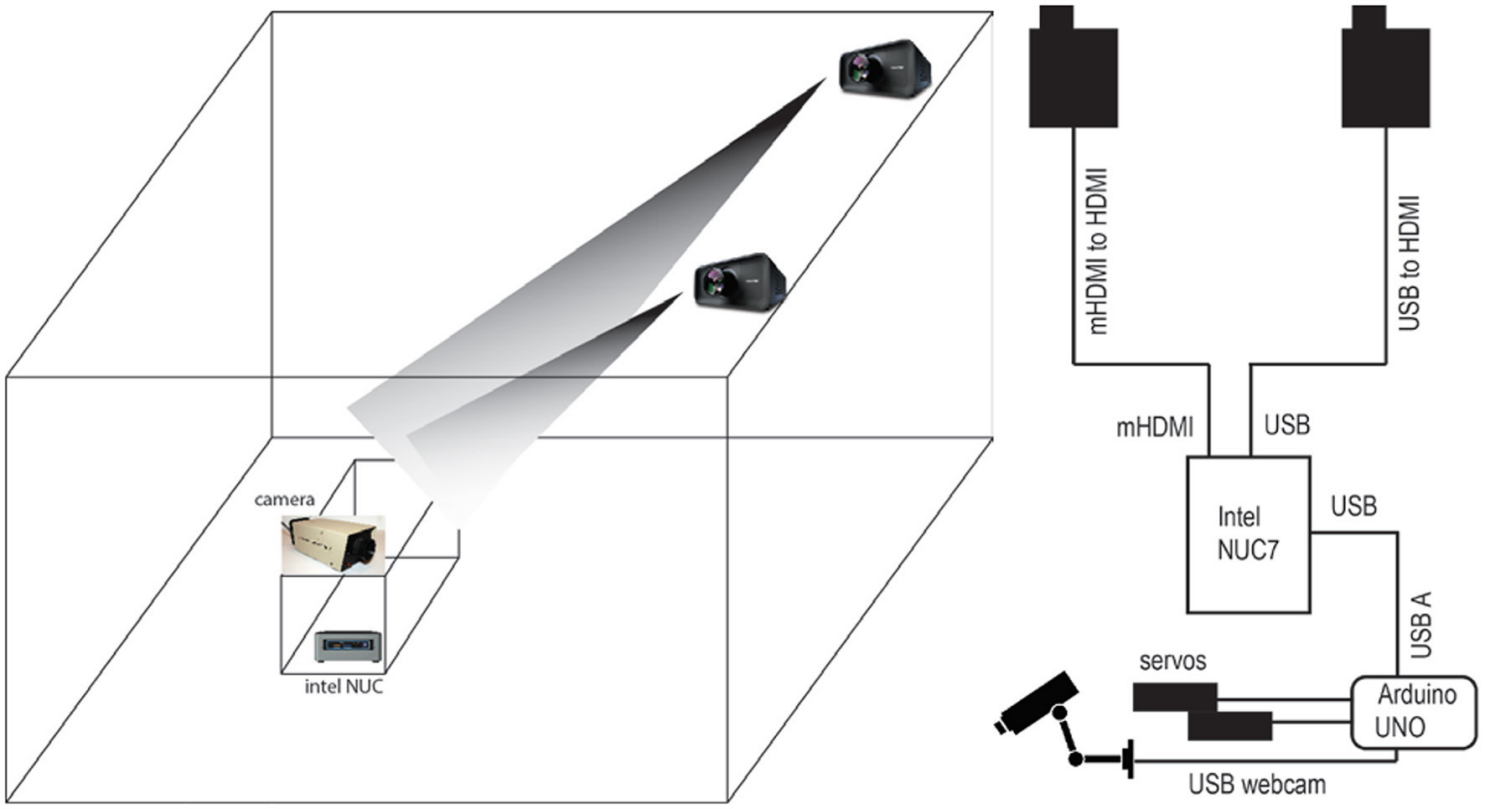
Exhibition plan. **(Left)** Projectors on railings were used to illuminate the face sculpture in the setup, while the NUC computer and motor board components were hidden in the inside of the cabinet. **(Right)** The connection diagram shows the NUC PC as the controller that integrated webcam input to decide whether to project a database face or a real face, and to direct the servo motors via arduino UNO how to move to keep the audience's face in the center. In other situations, it directed the robot to pan and tilt in a preprogrammed manner.

A set of prototypes for the 3D face sculpture were made using different media, in order to investigate how well projection mapping works given the current lighting situation at the museum. We tried clay, paper mache, PLA (3D print), a mushroom-based polymer, and foamular (CNC). Figure 2 shows two attempts in sculpture construction. We decided ultimately to work with foam due to the ability to scale up in size, the lighter weight of the material, the ability to precisely craft the 3D look of the sculpture using CNC, and its ability to reflect the projection imagery properly upon being painted. A 3D face model was constructed in Cinema4D, and one half of the face was transformed using the poly effect to look pixelated with large polygons. Thus, the two sides of the face looked slightly different under projection of a face, with one side appearing more digitally manipulated than the other. The models were converted to stl format and printed on a 48 × 32 × 8 inch foam. The face was painted white to allow projection image to reflect, while the rest of the foam was painted black and mounted on a dark-colored podium (Figure 3). Canon LV8320 (3000 lumens) projectors were used to project face images from a ~40° angle above the setup (Figure 4). The image was projection mapped onto the face sculpture and controlled from the NUC 7 computer using the Kantan Mapper module from Touch Designer v099.

**Figure 5:**
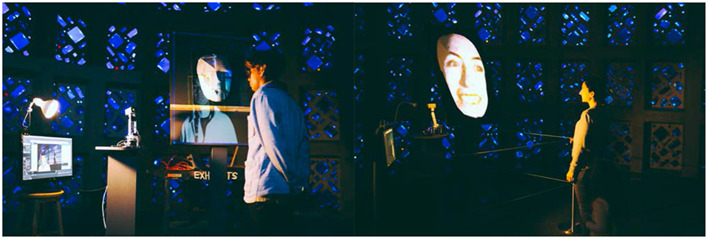
Audience interaction with the exhibit in prototype and finished form. **(Left)** Prototype stage interaction using a smaller face sculpture and brighter lamp to facilitate computer vision processes. **(Right)** A time during the final exhibition where the audience's face was detected, scaled, and projected onto the face sculpture. The projection mapping ensured the audience's face would be imaged on the face section of the sculpture. The audience's face, as seen from the robot's position, was shown on the screen to the left. At this stage, the robot followed the audience's face as it moved in space, as long as it was detected. When faces were no longer detected, the projection changed to flipping through the Chicago Face Database.

Completed views of the main interaction area are shown in Figure 3. The camera-mounted robot sat at the left of the projected sculpture. To its left was placed a live-view screen that showed the audience what the camera saw. When no faces were detected, the projection looped through a set of faces from the Chicago Face Database (chicagofaces.org) as a default visual response while the robot scanned the room. When a face was detected, Processing scaled the subject's face to the size of the projected image on the sculpture and used Spout to send the live video stream to Touch Designer to project onto the sculpture. The robot could follow the audience face by rotating or tilting during this interval so the image displayed was always dynamic. The size of the face projected on the sculpture was always the same regardless of the audience walking forward or away due to the scaling done in Processing. The image resolution is thus lower when the audience is farther away from the robot. When a face was found, a yellow square was also shown on the screen to the left superimposed on the camera's view. The complete system is diagrammed in Figure 4, and shown in audience view in Figure 5, both in prototype and final exhibition forms. Ambient lighting in the exhibition hall was turned down so that the projected image could be seen. Unfortunately this reduces the reliability of the computer vision. Thus, two lamps were mounted, one for illuminating the side of the robot, the other for lighting the audience's face for proficiency of computer vision through the robot's webcam camera. The lighting was calibrated at the beginning of each day of exhibition (from May to September of 2019) to ensure optimal audience experience each day.

### Workshop

A workshop opened to participants of all ages was created and presented 5 times at New York Hall of Science (NYSCI) by members of the museum's Explainers Program. At least half of the participants at each workshop were under the age of 18. Each workshop had 7–9 laptops with the capacity for 10–15 participants. The workshop began by asking subjects to draw a face while focusing on features like eyes, nose, lips, and glasses, as an exercise. For the next 5 min, everyone showed their drawings to the crowd, and the workshop staff showed a computer-generated face from thisfacedoesnotexist.com, highlighting uniquely human features and discussing briefly how computers see human faces differently from us. We also outlined the main goal of the workshop to understand and play with the way machines see us. The next 5 min were spent getting a laptop setup and navigating a webpage that shows how poses can be detected by the computer vision on the webcam on the laptop. In this phase, participants could get out of their chair and move around to see how it affects the pose determination.

For the main part of the workshop (the remaining 25 min), we introduced how machines learn to recognize specific faces and how we can escape their detection, a fun activity for younger audiences. We showed audiences a custom script based on an existing p5 sketch we used to train a face classifier (https://editor.p5js.org/AndreasRef/sketches/BJkaHBMYm). First, the audience clicked a button repeatedly to take pictures of their faces with multiple samples. After training the program, we let the participants come in and out of the view of the webcam to verify that the machine learning algorithm has learned a representation of their faces. Workshop staff were available to fix any problems children had, but overall we were surprised by the amount of computer literacy displayed by the children.

**Figure 6:**
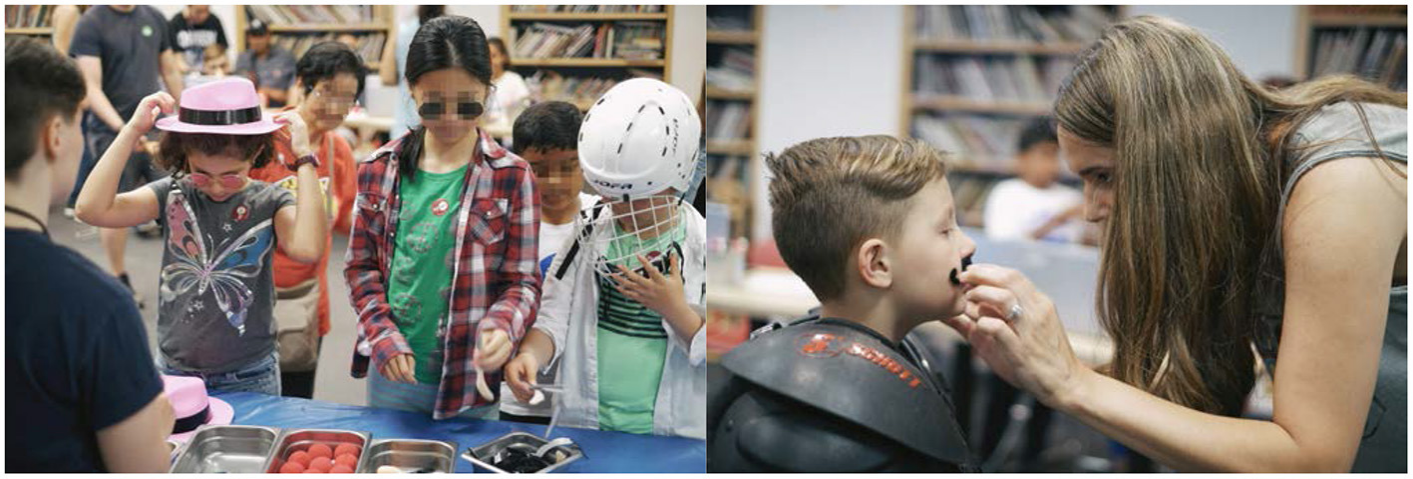
Workshop dress-up phase. **(Left)** Children selecting props, hats, decorations, and garments to wear that would allow them to escape the detection of a face classifier previously trained on their undecorated faces. **(Right)** A parent putting a fake mustache on her child after he put on football shoulder pads in an attempt to escape the computer vision's detection.

**Figure 7:**
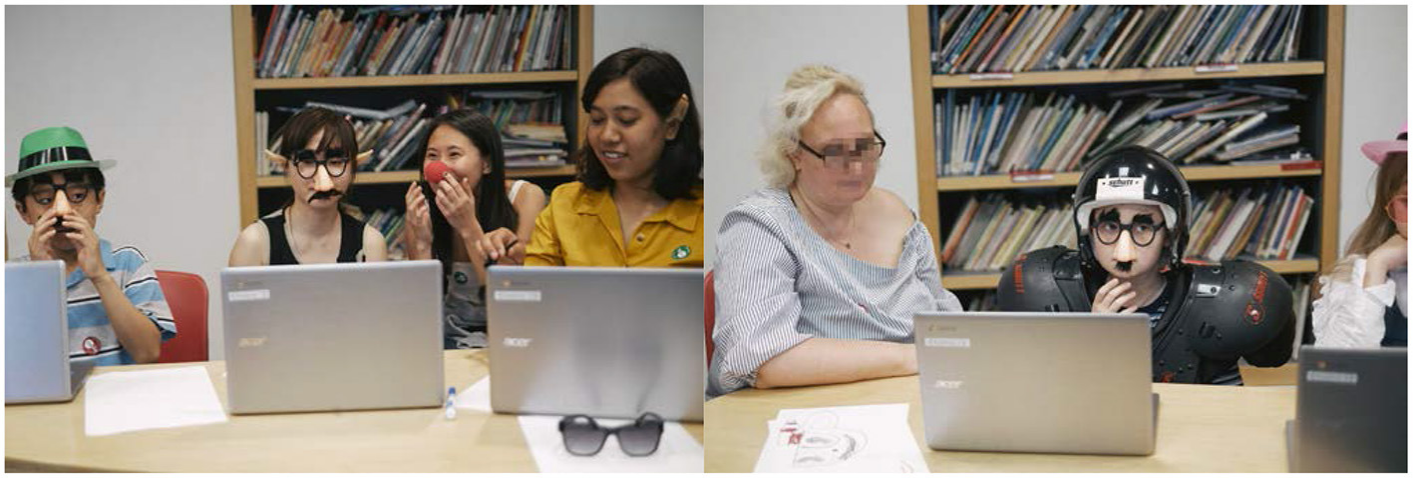
Workshop face-detection phase. **(Left, Right)** Children wearing disguises observing whether the p5 face classifier script running on the computer was able to distinguish between their real faces and their new disguises. Participants were able to vary the amount of disguises and how they were put on until the classifier detected them as unique faces.

Next we provided props like fake ears, hats, garments, mustaches, and jewelry to allow participants to dress up to escape the detection of the program despite being seen by the webcam (Figure 6). In this stage we showed how audiences can exist independently of the awareness of the machine. We let participants pick one outfit and train the program on the same person's face but as model for a different face. At this point, audiences could put on and take off their disguises and see the program recognizing different faces as different individuals (Figure 7). For example, one participant would train the program with his own face until it outputs “Danny” whenever his face is in front of the webcam. Then Danny would dress up as a football player and train the program to recognize the disguise as “Eli” (name of a well-known football player in New York). Then Danny would escape the program's detection of “Danny” by dressing up as Eli and vice versa. Throughout the process the workshop staff informed the participants on educational details about computer vision and machine learning. For example, we showed how taking many pictures (samples) were necessary for good recognition, the way different angles and conditions of a face for a given training made the algorithm more successful, and how these technologies were implemented in our own devices, etc.

**Figure 8:**
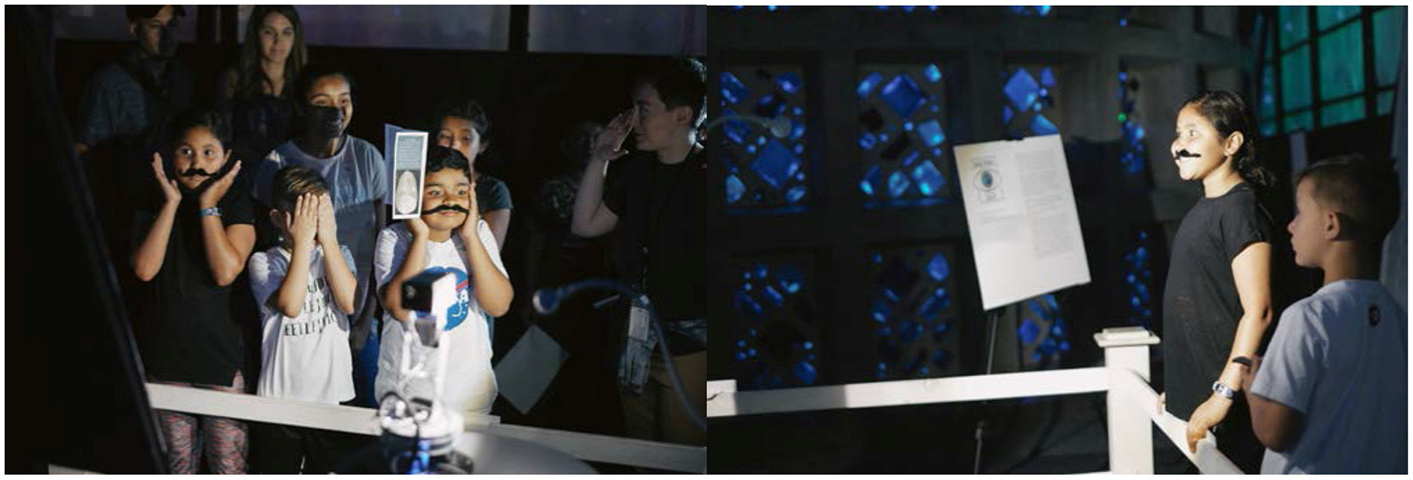
Workshop exhibition phase. **(Left, Right)** Children were ushered to exhibition after the workshop and allowed to explore interactions with “Machine Gaze.” They are currently looking into the robot's camera eye while also glancing to see if their face was detected by seeing whether their own faces appeared on the 3D face sculpture. Note that one child attempted to cover his face while looking through the slits between his fingers. The mustaches were left on by the children's choice.

**Figure 9:**
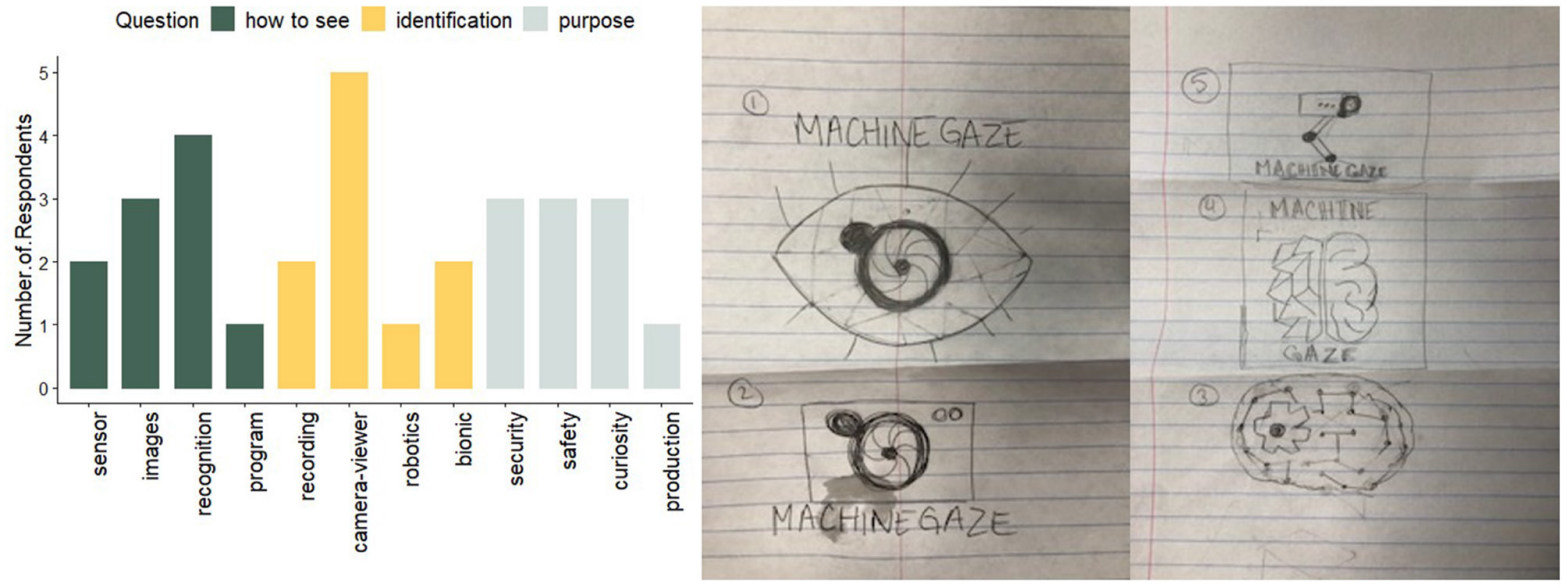
Audience experience during the exhibition. **(Left)** Distribution of coded answers to each pertinent question in the survey given after exhibition experience (*n* = 10) (see **Appendices** for raw questionnaire and coding process). (Green) Answers to “How do you think computers see us?” ranged from mentioning the camera's sensor abilities, by taking images, by recognizing people, and by using a computer program. (Yellow) Answers to “What do you think computer vision is?” ranged from computer as a recording device, to machine vision as a camera that views its environment, to robotics, to computer vision as a bionic device. (Gray) Answers to “What do you think the robot's purpose is?” included a role to protect security, a way to promote safety, as a curious machine, and for production of resource. **(Right)** Drawing by a young audience member that served as her interpretation of what the “Machine Gaze” exhibit meant to her.

After the workshop, we escorted the participants to the “Machine Gaze” exhibit (Figure 8), where they interacted with the robot and projected face sculpture freely for about 15 min before being given a questionnaire that asked the following 4 questions: “Where do you think the security camera comes from?,” “What do you think the robot's purpose is?,” “What do you think computer vision is?,” “How do you think computers see us?.” They were asked to answer in short phrases, which are coded qualitatively and presented (Figure 9). For a selected group of audiences, we followed the questionnaire with a qualitative interview to learn about their experiences in depth, asking them to elaborate on their reaction upon seeing their own image on the sculpture, how they managed to catch up with the robot's gaze when it stopped following their faces, how they interpreted their own image on the sculpture vs. what the machine sees (as shown on the screen), how they reacted to the machine moving between multiple faces being detected, where they allocated their attention when the displayed face switched from their own to that of another and vice versa, etc. The questionnaire answers were qualitatively coded into categories, tabulated and plotted in R 3.6.0. Finally, we passively observed audiences as they interacted with the exhibit, taking note of their tendencies, moments of joy, moments of confusion, and issues that arose. The interview questionnaire, and observation data were used to further refine the exhibit after the workshop ended and the main exhibition timeline began at NYSCI.

## Results

Production and prototyping of the exhibition is seen here: https://youtu.be/V42towEXruk. Note the discretized movements of the robot tracking movement in 0:28. We decided to keep the discretized movements after audience members indicated in the first item in the questionnaire that it made them feel like the camera was made long time ago in a “factory,” and would be found in the “corners in rooms.” The prototyping also showed that due to the OpenCV xml template used, even animal and cartoon faces were detectable (1:05), further allowing audiences to identify the machine's particular method of perception as something separate from human faculties. The initial face images we projected were also not uniform enough to suggest a set of possible machine perceptions, so we replaced them with the photos from the Chicago Face Database. Finally, we realized from preliminary interactions that the camera tended to move between multiple faces quickly in practice, so we set a delay of one second before it can move again during face tracking periods, so that audience members can see what's happening and react accordingly. Other materials/processes refined throughout the process included the material used to make the face sculpture, the lighting in the exhibition hall, the color of the podiums used, the speed of the robot movements, the number of projectors used, and size of the safety area around the robot, etc.

The full exhibition took place from May to September, 2019, with workshops kicking off the schedule in May. Documentation of audience interactions is here: https://youtu.be/kVoqkzZT4IQ. Our observation of the audience yielded three types of participants: (1) those curious about the device but refraining from making excessive contact with the machines (0:40), (2) those who take an active role to make expressive faces in engaging with the system (1:15), (3) those who bring others to the interaction by inviting them to the exhibit or enabling them to be in the view of the robot, creating a multi-face interaction (1:00). From our 5 days of observation, type (2) were the most numerous, with type (3) close behind, and perhaps exceeding type (2) on Sundays (the only weekend day we were observing). Interestingly, we found that group (1) audiences tended to come back to the exhibit at multiple points during their visits. One possible reason is that they interpreted the machine as standing guard over the exhibit, and hence came back to see if the machine would be off its guard (i.e., during periods where it stopped tracking faces). Group (2) audiences tended to make interesting discoveries in their interactions, such as using their hands to cover their faces so that the machine cannot see them (but they can see the machine move), and other pictures, people, and instruments in the environment as bait for the machine to focus its gaze on. Group (3) audiences included many parents who took their children in their arms while exploring the interaction together. They tended to initially guide the child's discovery, but frequently ended up competing with them for the machine's attention.

The audience survey given after workshop interactions showed different audience perceptions that we were initially unaware of Figure 9. While most participants equated computer vision with some sort of camera-seeing process (see yellow bars in Figure 9), some were associating it with recording or human-augmentation, topics with which computer vision is associated in popular culture. As in previous work, audiences tended to assign machine intelligence to the robot system beyond simple mechanical processes. In answer to how the robot sees, most participants attributed its ability to some recognition capability beyond simply sensor-reading or photography. We were also surprised to see that 3 of the 10 audience members surveyed also attributed the purpose of the machine to its curiosity or need for discovery, an inherently non-mechanical goal that assigns a human-like emotional content to the machine. People appear to be attributing advanced technologies to an old supermarket camera, assigning more intelligence to it than expected based on appearance, analogous to previous works in the area (Sirkin et al., 2015; LC, 2019).

However, due to the small number of participants (*n* = 10) and the free-form nature of the responses, we must warn against over-interpreting the data. Future work will be needed to tease out audience perceptions in complex mirror-like machine interactions, including with devices that perform only the mirror projection part, or only the machine looking-reflecting part. After the workshop, one artistic audience member drew some prospective logos for us. Her drawings equated the shutter of the camera to the human eye, and its hardware with the human brain, again assigning anthropomorphic qualities to the machine. We believe this reaction is due to the ability of the machine to move in space, indicate emotions like curiosity, aversion, boredom, intelligence, and attention through movement and changes in projected content. This may drive a sense of the audience feeling perceived by a being aware of the audience's persistence. It also hints at the use of robotics as a performance experience in evoking audience reaction.

Interactions from the workshop are shown here: https://youtu.be/pIRETXKZngg. Analysis was done on the videos after the workshop. For the face training phase, we saw that audiences liked to work as teams, usually with one member of the team (such as the parent) driving the interaction. Participants became creative with their interactions, such as turning around, glancing from beneath the table, and moving their face from side to side (0:48) as many ways to test the limits of escaping machine detection. We also observed parents teaching children about what it means to see their own image and how the machine interprets the face image (0:53). During the disguises section, we saw that the most popular items were hats (1:02). Frequently the participants helped each other put on the costumes and props and showed a feedback loop of asking for an opinion, then rearranging the props, and asking for opinion again, as if the questioner was using the opinion as a proxy for a mirror. Outlandish costumes were observed as well (1:11), because some faces did not easily escape the face detection algorithm, necessitating extreme measures. Interestingly, family members would sometimes wear matching outfits (1:18). This may be an indication of in-group affinity, but it could also indicate one member of the family teaching the other which disguises appear to be working. Generally the workshop was highly collaborative, with families working together and learning together. Finally, children tended to keep part of their disguises while visiting the exhibit (1:32). There was usually great excitement when seeing their own (disguised) faces appear on the 3D face sculpture, indicating their own shift in identity was registered by the perceiving system as well.

## Discussion

This intervention attempts to show audiences how perception of machines gazing at themselves can be a tool to engender self-awareness as a collaborative performance between human and machines. A first hint of these developments comes with the games that children invent while they interact with the robot. As detailed in Results, participants spontaneously perform games like covering their faces with their hands, making funny faces, seeing which of two faces the robot turns toward, etc. All these actions have a manifestation in the projected image on the 3D sculpture, some changing the detection of their face (covering with hands), some not changing the detection interaction (funny faces). The spontaneous development of these performative behaviors suggests an underlying learning process whereby children (and adults) acquire knowledge about whether they'll be perceived by the robot system based on the different performances they make. Their reaction to whether they are detected or not suggests an understanding of what the machine sees and how that relates to their concept of self. This understanding also seems to develop over the course of the interaction, with lack of understanding at first, followed by recognition of the machine gaze, then understanding of how they are perceived, and finally what they can perform to modulate this perception. To further test this idea, additional study is necessary to separate the self-identification process from the machine-perception process, and analyze how perception of each process emerges from interaction in the exhibit.

A second hint comes from the consistent attribution of human-like emotion, agenda, and behaviors to machines by audiences despite observing merely simple gestures, as previously studied (LC, 2019). The post-visit questionnaire results and exhibition audience observations both show some degree of assigning of human-like characteristics to the machine. For example, the machine is deemed to be curious by a large contingent of observers, and subsequent drawings of the machine endow it with human characteristics like eyesight. Audiences often treat the machine like a human-like creature both while it tracks their faces and when it ignores their faces. In the former they play movement games with it; in the latter they try to get its attention by moving toward the machine's eye voluntarily. This demonstrates that not only can machines track the human face, the human can track the machine face as well while trying to get its attention. This then creates a bi-directional interaction: if the audiences can see their own faces when the machine follows them, does the machine see its own face when they follow it? Further research beyond art interventions will be necessary to establish how these internal models about how each entity observes and is aware of itself may be able to provide educational moments for the participants themselves. Here, we propose through qualitative observation of audience interaction with an exhibition that such more complex dynamics involving processes of observing and modeling how machines see may be part of audience engagements.

A third hint comes from workshop interactions, where participants specifically escape detection of the machine's gaze by dressing up as another. The dressing-up serves as a narrative approach to differentiating who one is and is not (Bamberg, 2011), showing the actors who they are by letting them experiment with a situation where they are not perceived. This escape of detection may be critical in the audience members' self-concept, for they are able to recognize that sometimes they won't be perceived by others if they only performed a certain way. It's as if audience members are playing a game of public performance akin to self-presentation that hides their own true identities in the context of robots and environments that are not sophisticated enough to understand this form of deception. More interventions will be necessary to show how these mini-deceptions and playful performances affect what participants think of themselves in the context of environmental modulations.

## Conclusions

Children's perception of being seen or not seen by external entities like mirrors and other people helps define their self-awareness. This identity is associated with their own self-presentation, which forms a performative behavior in public that in turn reinforces who they should be (Goffman, 1959). In this artistic intervention, we created a mirror-projection system that shows audiences their own faces, but only when interaction requirements are met, so that their perception of themselves are framed by what a machine sees, a form of performance in spatial interaction. We leveraged the prior demonstration of effectiveness in using robotics to help socialize children with communication disorders like autism (Boucenna et al., 2014) to create embodied physical actions that transform simply passive viewing to interactive behaviors that capture the subtleties of a self-perception-dependent form of performance. As audience interactions and experience shows, the exhibit and accompanying workshop leave participants more aware of how machine perception works, how their own actions interact with these perceptions, and how their own performance with the machines engenders cooperative awareness of the limitations of each.

The use of environmentally enriched robotic interactions is promising in artistic and social design realms, both for treating those with communication issues and for creating interactive experiences for the general public. This exhibition showed one possible intervention in provoking audiences to examine what their self is by using physical embodied interactions with a computer vision-enabled camera that detects their face. In the workshop, we showed that intervention contributes a sense of self identity for children, while in the exhibition, we argue that the distancing away from self-benefits audiences of all ages by allowing them to see themselves through eyes of the other. These technologies provide possible future scenarios of more intimate interactions that takes into account more affective types of human data beyond face detection. This work suggests a future direction toward smart environment and robotic interactions that can leverage human psychological insights to design interventions that aim to push for societal good.

## Data Availability Statement

The raw data supporting the conclusions of this article will be made available by the authors, without undue reservation.

## Ethics Statement

Ethical review and approval was not required for the study on human participants in accordance with the local legislation and institutional requirements. Written informed consent for participation was obtained from the participants or their legal guardian/next of kin. Written informed consent was obtained from the participants or their legal guardian/next of kin for the publication of any potentially identifiable images or data included in this article.

## Author Contributions

RLC, AA, AB, and ST created the exhibition. RLC and ST produced the figures. AA, AB, and ST ran the workshops and collected the data. RLC wrote the manuscript. All authors contributed to the article and approved the submitted version.

## Conflict of Interest

The authors declare that the research was conducted in the absence of any commercial or financial relationships that could be construed as a potential conflict of interest.

## APPENDIX A

Audience Questionnaire given after the workshop.

Thank you today for participating in the Machine Gaze workshop at NYSCI. Please answer the following to the best of your abilities with a short answer (one phrase only).

1. Where do you think the security camera comes from?
2. How do you think computers see us?
3. What do you think computer vision is?
4. What do you think the robot's purpose is?

A BIG thank you from the explainers and staff of NYSCI. Hope you had fun, and keep playing with technology here at the museum today.

## APPENDIX B

Examples of coding of responses to survey questions.

**Table:**

**Question full**		**Raw responses**								
How do you think computers see us?	Little thing in the camera	Camera	Black and white images	Code (zeroes and ones)	Recognize us as shapes and images	Recalling things	Camera takes a picture during video	Through the lens	By figuring out where we are	Tracks our faces
CODE	SENSOR	IMAGES	IMAGES	PROGRAM	RECOGNITION	RECOGNITION	IMAGES	SENSOR	RECOGNITION	RECOGNITION
What do you think computer vision is?	Looking through a vision	Camera	Microscope	Machine lens you see (you see through a machine lens)	You look at something too long that it starts to affect you.	Recording	Robots	Camera lens	Machine for eyes (machine or bionic eyes)	Video recorder
CODE	CAMERA-VIEWER	CAMERA-VIEWER	CAMERA-VIEWER	CAMERA-VIEWER	BIONIC	RECORDING	ROBOTICS	CAMERA-VIEWER	BIONIC	RECORDING
What do you think the robot's purpose is?	Looking from the corner	Making things in factory	Security, spy, prevent thief	Hidden security cameras	To see what happens around you	Camera makes sure nothing goes wrong	Makes sure people are safe	It's just looking around	Make sure everyone is ok	Monitor the crowd
CODE	CURIOSITY	PRODUCTION	SECURITY	SECURITY	CURIOSITY	SAFETY	SAFETY	CURIOSITY	SAFETY	SECURITY
