# Supplementary figures and images for: Machine Gaze: Self-Identification Through Play With a computer Vision-Based Projection and Robotics System
Source: Front Robot AI. 2020 Dec 17;7:580835. doi: 10.3389/frobt.2020.580835 (PMC7805933; doi:10.3389/frobt.2020.580835)

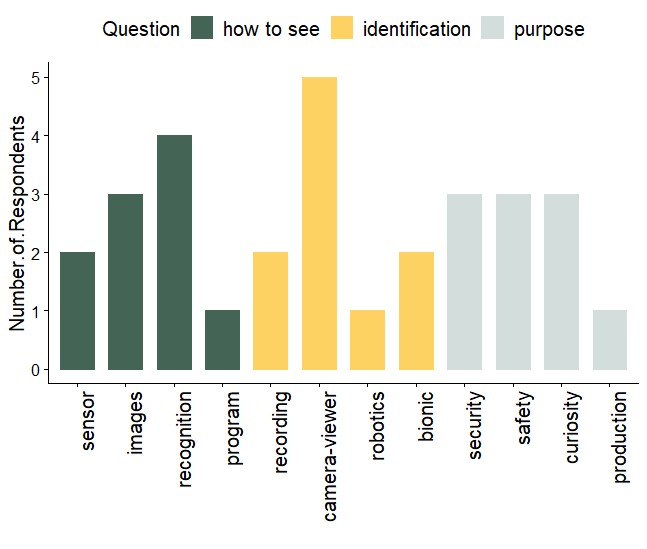

Supplement: Supplementary file 1 [file Image_1.JPEG]

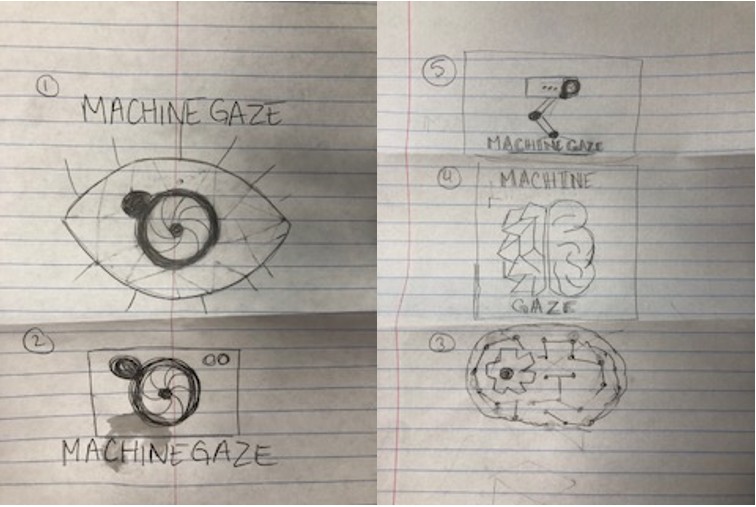

Supplement: Supplementary file 2 [file Image_2.JPEG]

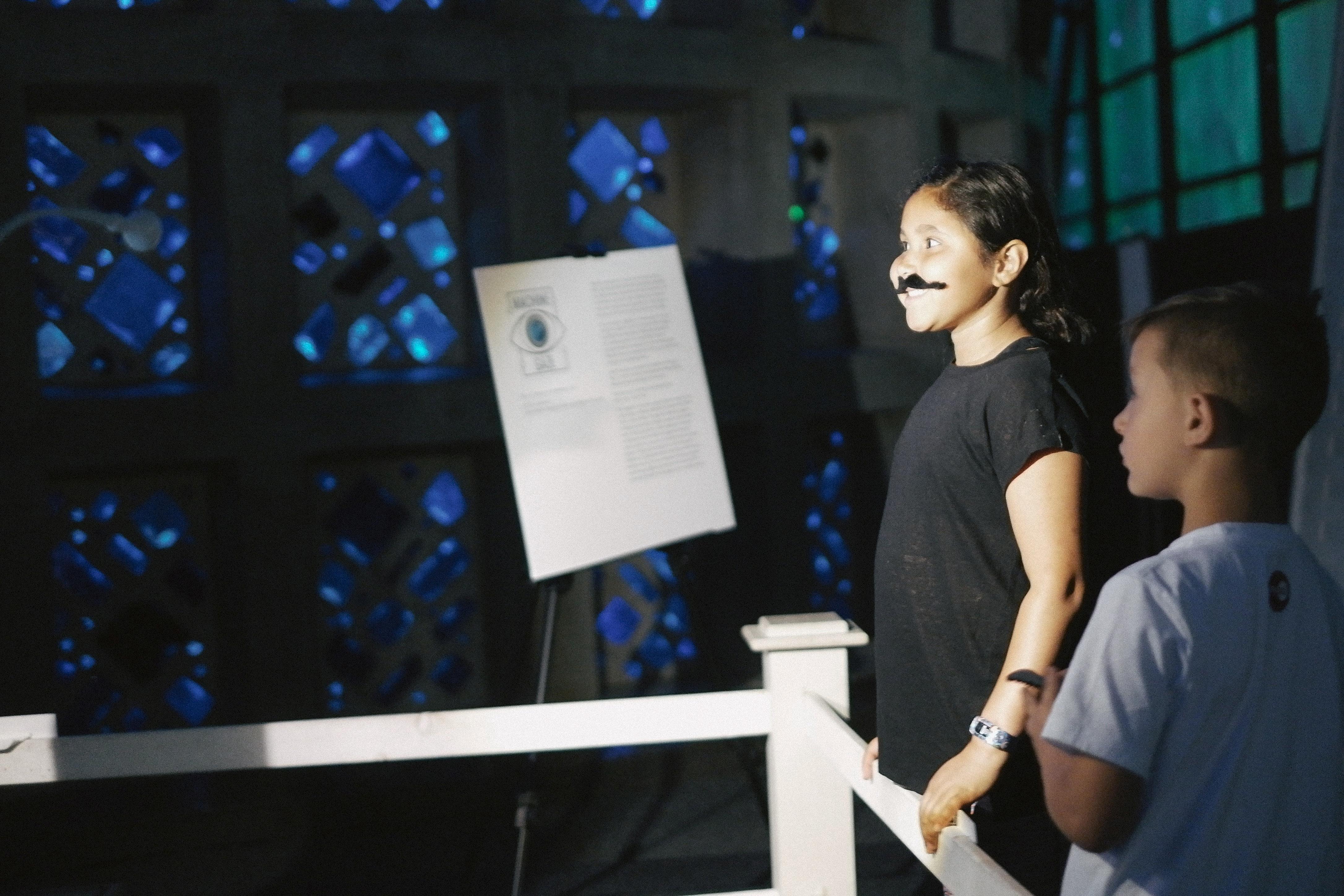

Supplement: Supplementary file 3 [file Image_3.JPEG]

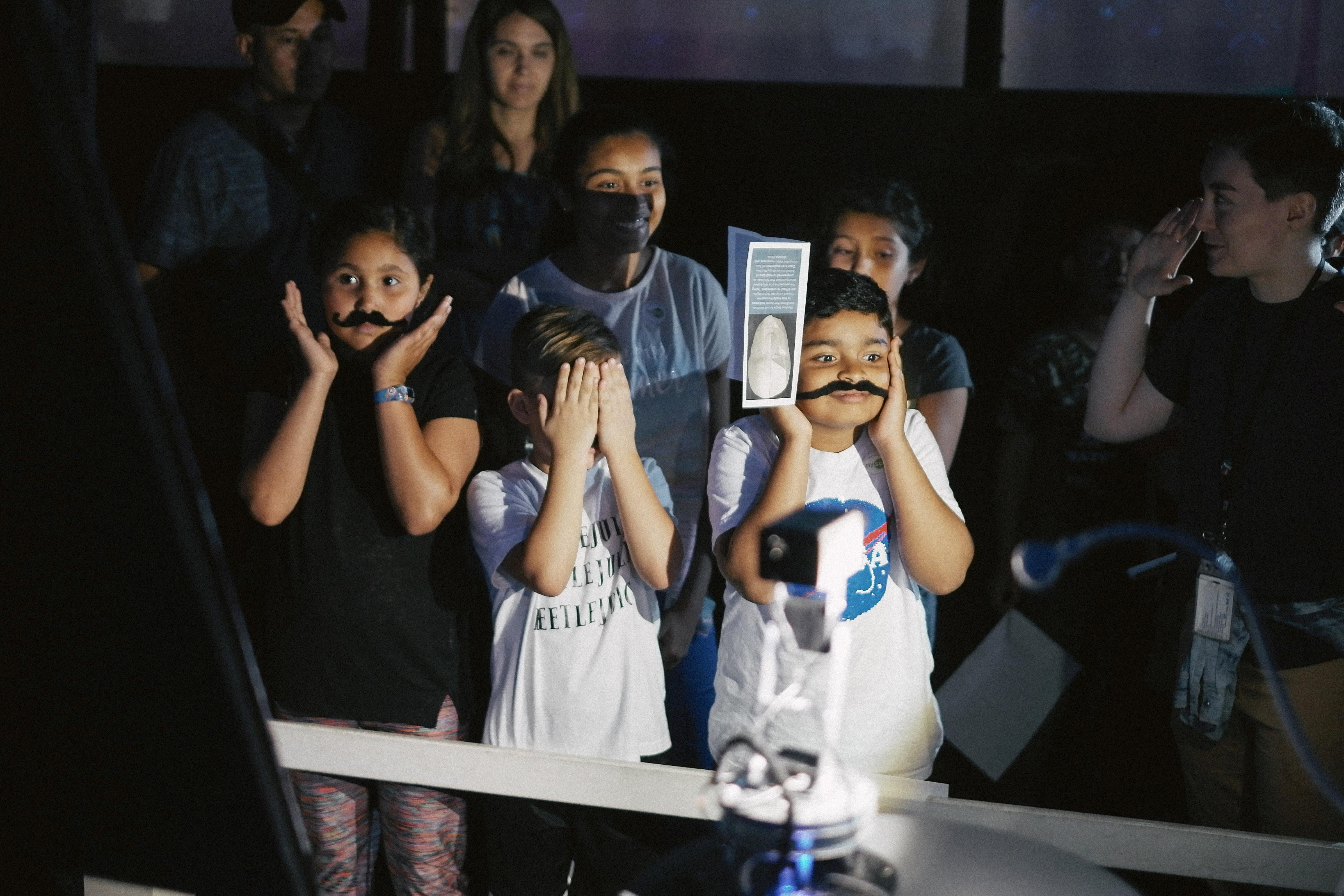

Supplement: Supplementary file 4 [file Image_4.JPEG]
